# Activation of Perovskite Nanocrystals for Volumetric Displays Using Near-Infrared Photon Upconversion by Triplet Fusion

**DOI:** 10.3390/molecules30112273

**Published:** 2025-05-22

**Authors:** Yu Hu, Guiwen Luo, Pengfei Niu, Ling Zhang, Tianjun Yu, Jinping Chen, Yi Li, Yi Zeng

**Affiliations:** 1 Key Laboratory of Photochemical Conversion and Optoelectronic Materials, Technical Institute of Physics and Chemistry, Chinese Academy of Sciences, Beijing 100190, China; huyu20@mails.ucas.ac.cn (Y.H.); niupengfei18@mails.ucas.ac.cn (P.N.); zhangling21@mails.ucas.ac.cn (L.Z.); tianjun_yu@mail.ipc.ac.cn (T.Y.); chenjp@mail.ipc.ac.cn (J.C.); yili@mail.ipc.ac.cn (Y.L.); 2 University of Chinese Academy of Sciences, Beijing 100049, China

**Keywords:** triplet–triplet annihilation, upconversion, perovskite quantum dots, near-infrared, light-harvesting

## Abstract

Coupling organic light-harvesting materials with lead halide perovskite quantum dots (LHP QDs) is an attractive approach that could provide great potential in optoelectronic applications owing to the diversity of organic materials available and the intriguing optical and electronic properties of LHP QDs. Here, we demonstrate energy collection by CsPbI_3_ QDs from a near-infrared (NIR) light-harvesting upconversion system. The upconversion system consists of Pd-tetrakis-5,10,15,20-(p-methoxycarbonylphenyl)-tetraanthraporphyrin (PdTAP) as the sensitizer to harvest NIR photons and rubrene as the annihilator to generate upconverted photons via triplet fusion. Steady-state and time-resolved photoluminescence spectra reveal that CsPbI_3_ QDs are energized via radiative energy transfer from the singlet excited rubrene with photophysics fidelity of respective components. In addition, a volumetric display demo incorporating CsPbI_3_ QDs as light emitters employing triplet fusion upconversion was developed, showing bright luminescent images from CsPbI_3_ QDs. These results present the feasibility of integrating organic light-harvesting systems and perovskite QDs, enabling diverse light harvesting and activation of perovskite materials for optoelectronic applications.

## 1. Introduction

Lead halide perovskite quantum dots (LHP QDs) are an intriguing member of perovskite materials with large absorption cross-sections and narrow-band emission [1,2,3,4,5]. The unique optoelectronic properties and superior luminescence quantum yields with the defect tolerant feature, as well as the relatively easy preparation by varying LHP QDs composition and size, enable LHP QDs extensive applications in light-emitting diodes, photovoltaics, and photochemical conversion fields [3]. LHP QDs have a significant role in light-harvesting and sensitizing, achieving energy transfer from excited LHP QDs to organic species [6,7,8,9]. However, when focusing on their application in light-harvesting, it is found that the intrinsic absorption threshold (<700 nm) of perovskite quantum dots limits the capture of near-infrared (NIR) photons, thereby hindering their further applications [10]. Utilizing the full spectrum of solar energy or being responsive to near-infrared light is very attractive for optoelectronic device research, be it organic devices or perovskite materials [11,12,13]. An upconversion approach can enable the capture of NIR photons indirectly [14,15]. Among the various upconversion approaches, the lanthanide-doped nanoparticles or two-photon absorption techniques face the challenge of high optical excitation power density (Table S1). Referring to LHP QDs as energy acceptors, Chen has recently developed LHP QDs with tunable emission throughout the visible region sensitized by lanthanide-doped nanoparticles [16]. Using LHP QDs to capture near-infrared (NIR) photons via energy transfer has potential in optoelectronic devices [10]. Nevertheless, to apply the lanthanide-doped system further, the barriers of high excitation power density and low upconversion quantum yields need to be addressed [17]. Given their strong band-edge transitions and high emission quantum yields, LHP QDs can be used as singlet energy collectors to harvest upconverted photons produced by triplet fusion upconversion of organic compounds. This approach can simultaneously exploit the diverse functionality and excitonic energy of organic compounds and the favorable optical and electronic properties of LHP QDs to expand optoelectronic applications [10]. Meanwhile, this system enables the creation of volumetric displays by utilizing the strong monochromaticity of QDs and the triplet fusion upconversion system’s high upconversion quantum yield in a synergistic manner [18,19]. Triplet fusion upconversion via triplet fusion of two organic triplets is generated by sensitization [20,21,22,23,24,25], which captures and converts photons below bandgaps of conventional semiconductors to higher energy ones Seunder the non-coherent light source of low power density, holding the potential to augment photovoltaic [26,27,28] and photochemical conversion [29,30,31,32,33,34,35,36,37]. Singlet back energy transfer is highly susceptible to occur due to the more severe spectral overlap between the acceptor and the sensitizer in the triplet fusion upconversion system. And recently, organic singlet energy collectors have been introduced to harvest singlet excitons and enhance the upconversion performance [38,39,40,41,42]. The narrower emission spectrum of the quantum dots exhibits less spectral overlap with the absorption spectrum of the sensitizer, which reduces energy reabsorption by the sensitizer due to fluorescence resonance energy transfer.

Here, singlet energy harvesting of upconverted photons from organic upconversion systems is achieved by using CsPbI_3_ QDs. The triplet fusion upconversion component absorbs 808 nm NIR photons and upconverts to yellow ones, which transmits energy to LHP QDs by a radiation mechanism, thus realizing NIR light-illuminated LHP QDs luminescence. The upconversion system used in this study can operate at a power density as low as 134 mW/cm^2^. In addition, the organic system facilitates the upconversion quantum yield of QDs luminescence to reach 0.48% upon capturing NIR photons. This study establishes the approach using triplet fusion upconversion and other organic light-harvesting materials to energize LHP QDs with NIR photons.

## 2. Results

### 2.1. Characterization of Photophysical Properties

**Figure 1:**
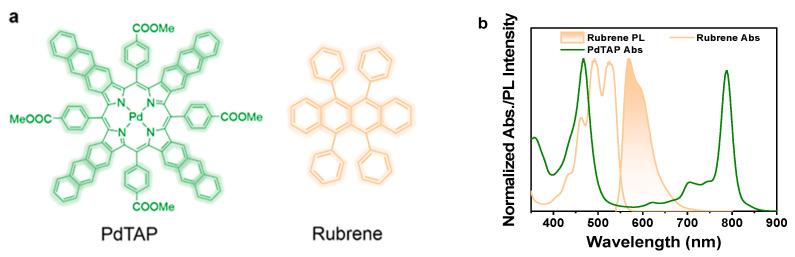
(**a**) Molecular structures of PdTAP (sensitizer) and rubrene (annihilator). (**b**) Normalized absorption and emission spectra of rubrene (orange) and PdTAP (green) in dilute toluene solutions (λ_ex_ = 530 nm).

The triplet fusion upconversion organic system consists of palladium(II) tetrakis-5,10,15,20-(p-methoxycarbonylphenyl)tetraanthraporphyrin (PdTAP) and rubrene as the sensitizer and the annihilator, respectively (Figure 1a). The sensitizer PdTAP functions through harvesting low-energy photons, intersystem crossing into its triplet state, and sensitizing the annihilator rubrene. Two rubrene triplets encounter and annihilate within their lifetime, generating one ground-state rubrene and one excited singlet rubrene. The latter emits photons of higher energy than those absorbed by the sensitizer. PdTAP can be excited by NIR photons in its Q-band absorption region and then effectively convert to its triplet state of about 1.14 eV, estimated from the phosphorescence peak (Figure S5). As previously reported [43], the triplet state of rubrene is estimated to be at 1.14 eV, making it a suitable annihilator to be sensitized by PdTAP. In addition, the luminescence of rubrene has minimal overlap with the absorption band of the sensitizer PdTAP, which avoids the back energy transfer caused by reabsorption (Figure 1b). Therefore, using rubrene and PdTAP as the donor–acceptor pair is anticipated to construct a suitable NIR upconversion system.

**Figure 2:**
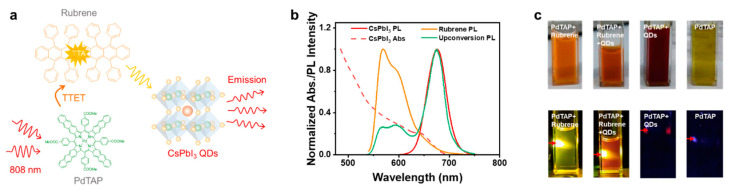
(**a**) Illustration of NIR energy collection by LHP QDs from the organic upconversion system. (**b**) Normalized absorption and emission spectra of rubrene (orange) and CsPbI_3_ QDs (red) in dilute toluene solutions. The green line is the upconversion luminous spectrum of this system. (**c**) Photographs were taken under four comparative experimental conditions. The upper and lower photographs are taken under natural light and excitation at 808 nm, respectively, and the red arrows in the photographs indicate the direction of incident light. All photographs were taken without bandpass filters.

The organic upconversion system was prepared by adding PdTAP into rubrene solutions at a molar ratio of 1:20, ensuring ample free annihilators. The photophysical properties of PdTAP-doped rubrene solutions (PdTAP/rubrene) were established in an oxygen-free environment, as described in Section 3 Upon excitation with an 808 nm continuous-wave (CW) laser, PdTAP/rubrene systems display upconverted yellow emission with the rubrene characters (Figure 2c). As the excitation power density increases, the upconversion emission intensity increases and presents a typical transition from quadratic to linear dependence on the excitation power density, confirming that the upconversion emission is generated via triplet fusion. Two linear fits are obtained with slopes of 2.20 and 1.17 in the low and the high excitation power density regimes, respectively, and the extrapolated intersection of the lines estimates the threshold excitation power density (Ith) to be 2.95 W/cm^2^ (Figure S11). Ith is a parameter indicating the initial transition between quadratic and linear excitation power dependence, where half of the triplet annihilators are involved in the triplet fusion. Below Ith, the spontaneous decay of the triplet annihilator dominates, while above Ith, the triplet fusion process surpasses the spontaneous decay resulting in linear excitation power dependence of the upconverted emission, and the triplet fusion process starts being saturated [44,45]. The excitation intensity threshold (Ith) can be improved by optimizing the excitation wavelength (785 nm) of the sensitizer [43,46]. The upconversion quantum yield of PdTAP/rubrene solutions was measured to be 1.03% (with a theoretical maximum quantum yield of 50%) under an excitation power density of 13.1 W/cm^2^.

CsPbI_3_ QDs were prepared according to the literature [47] and were designed to serve as the upconversion emission collector. The cube-shaped CsPbI_3_ QDs with an average size of 8.2 nm (Figure S6) show the first excitonic peak at 642 nm, which matches the fluorescence of rubrene (Figure 2b), indicating the potential for efficient energy harvesting. The CsPbI_3_ QDs in toluene exhibit a photoluminescence (PL) quantum yield of 60% and a maximum peak at 676 nm with a linewidth of 44 nm (Figure S7). The PdTAP/rubrene solution was mixed with CsPbI_3_ QDs at concentrations ranging from 0.05 to 1 mg/mL, forming the upconversion combination of PdTAP/rubrene/QDs. Activation of CsPbI_3_ QDs by triplet fusion upconversion was initially examined through steady-state emission spectra upon excitation at 808 nm (Figure 2c and Figure S9). When QDs were incorporated, the time-resolved luminescence spectra exhibited negligible change, indicating the absence of influence of QDs on triplet energy transfer (TET) in the triplet fusion upconversion system (Figure S8).

No emission was detected from CsPbI_3_ QDs upon excitation with 808 nm CW laser up to 2 W/cm^2^, since there is no linear absorption or multiphoton excitation at such excitation conditions. The slope change in the upconversion luminescence intensity with power density shows a typical dependence, indicating that the luminescence of QDs originates from the upconversion system. Additionally, the QDs do not change the threshold of the excitation power density of the original system, which indicates that the QDs do not affect the photophysical process of triplet fusion. Power density-related tests also show that the intensity of the NIR light source required to activate this hybrid system is only 134 mW/cm^2^, which is over 22 times less than the reported value (Figure S11, Table S1) [17].

The total upconversion emission quantum yield for the PdTAP/rubrene/QDs was also measured in the same way as that of PdTAP/rubrene, giving an upconversion quantum yield of 0.48% under an excitation power density of 13.1 W/cm^2^. Taking into account the luminescence quantum yield of QDs, the decline in value in comparison to the previous PdTAP/rubrene system stems mainly from a result of the comparatively low luminescence quantum yield exhibited by the CsPbI_3_ QDs.

**Figure 3:**
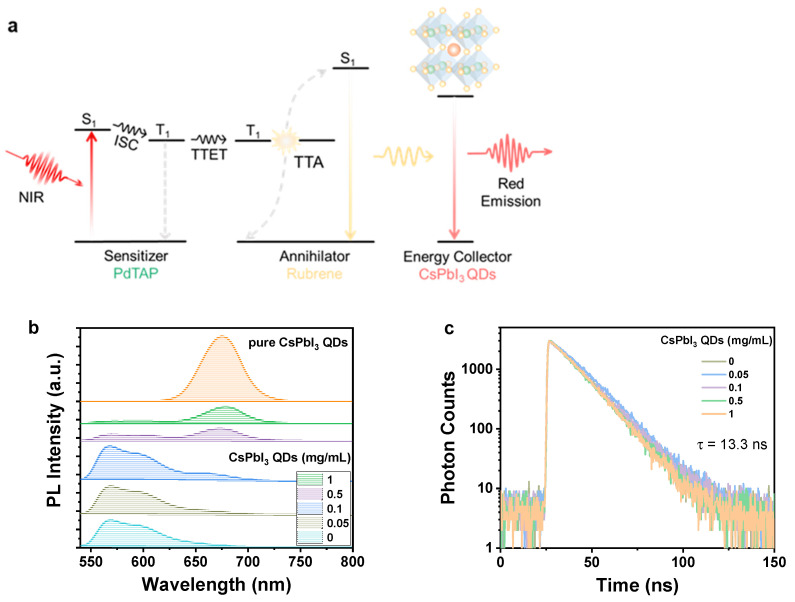
(**a**) Schematic illustration of NIR photons harvesting by perovskite QDs via triplet fusion upconversion. (**b**) Emission spectra of rubrene doped with different concentrations of CsPbI_3_ QDs in toluene solution (λ_ex_ = 530 nm) together with the PL spectrum of pure QDs for comparison. (**c**) Decay traces of the rubrene fluorescence at 550 nm in the presence of different concentrations of CsPbI_3_ QDs (λ_ex_ = 510 nm).

Figure 3b shows that rubrene luminescence is gradually decreased with the increasing QD concentration. Based on the sufficient spectral overlap (*J* = 2.71 × 10^−10^ M^−1^ cm^3^) between rubrene and QDs, it is proposed that the energy transfer from rubrene to QDs mainly accounts for the decrease in the upconversion fluorescence of rubrene. To confirm the energy transfer from rubrene to CsPbI_3_ QDs, the excitation spectra of the mixed solutions of QDs and rubrene were measured and shown in Figure S10 together with those of pure QDs and rubrene solutions. The excitation spectra of QDs mixed with rubrene show the typical excitonic band from the QDs around 660 nm and distinct peaks in the region of 550 nm where rubrene absorbs. The intensity of the excitation peaks with rubrene characters decreases with the increasing QDs content. These observations verify the occurrence of energy transfer from rubrene to the QDs. The energy transfer efficiency reaches 80% when the concentration of QDs is 1.0 mg/mL. Higher concentrations of QDs will ensure higher energy transfer efficiencies. However, higher concentrations of QDs make it difficult for visible light to pass through, which is unfavorable for display applications. Time-resolved emission spectra were further investigated to understand the mechanism underlying the energy transfer process. The fluorescence decay trace of rubrene solutions upon excitation at 510 nm was fitted monoexponentially, giving a lifetime of ca. 13.3 ns. The rubrene solution mixed with QDs exhibits photoluminescence decay overlapping with that of rubrene alone (Figure 3c), implying that there is no direct interaction between the excited rubrene and QDs. Furthermore, the delayed upconversion lifetime of the system does not change significantly after the introduction of the QDs (Figure S13). The data here demonstrate that the energy transfer from rubrene to the QDs proceeds via radiative energy transfer, otherwise the fluorescence lifetime or delayed luminescence lifetime of rubrene should be changed. Therefore, the photophysical process within the system is that the organic component absorbs NIR photons and produces upconversion luminescence, which is then absorbed by the QDs to reproduce luminescence (Figure 3a).

### 2.2. Volumetric Displays

**Figure 4:**
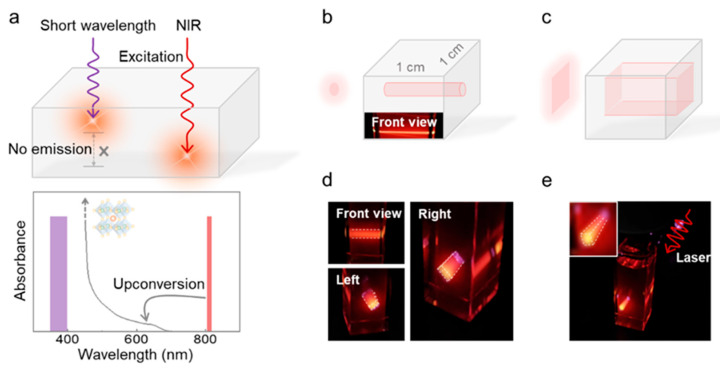
(**a**) Schematic illustration of the difference between short-wavelength and NIR excitation of QDs. The penetration of short-wavelength-excitation decreases significantly as the QDs content increases. (**b**) Diagram illustrating the projected cylindrical pattern. A haloed circle indicates the NIR light source. (**c**) Diagram depicting the projected representation of a cuboid pattern. A haloed rectangle indicates the NIR light source. (**d**) Photographs of the specific luminescence of the cuboid pattern taken from different angles in the dark environment. (**e**) Photographs of the specific luminescence of the quadrangular prism pattern in the dark environment. The inset shows a partial enlargement of the model. All photographs were taken without bandpass filters.

The activation of QDs by the rubrene fluorescence lays the foundation for upconversion activating QDs. NIR upconversion activating QDs provides a feasible way to overcome the transmission limitation and the direct excitation along the optical path, which is caused by intrinsic absorption coefficients in the visible range (Figure 4a). We have fabricated a square projected display demo using the above-mentioned mixture of perovskite QDs and upconversion materials. NIR light is used to illuminate this mixture and the luminescence of perovskite QDs can be observed, projecting the designed contour. Furthermore, the projected display can benefit from the photochemical conversion of low-power density excitation, requiring only a few hundred milliwatts of laser power. Circular and square light sources were used to excite the square samples, resulting in the observation of cylindrical (r = 0.5 mm, h = 10 mm) and cuboid (a = 3.5 mm, b = 1 mm, and c = 10 mm) perovskite quantum dots’ luminescence patterns as shown in Figure 4b–d. These observations were made under filter-free conditions. Controlling laser focusing enables the realization of a particular display pattern, such as a quadrangular prism (Figure 4e). The different alignments of the luminescent patterns show the innate emission of the QDs and accentuate their three-dimensional property traits. The stability of the system upon irradiation was also evaluated. The absorption spectra and the emission characteristics remain stable under continuous laser irradiation over a 30 min period (Figure S14). In addition, due to the quadratic dependence of triplet fusion upconversion on the excitation power density, the samples are capable of achieving a point display with sub-mm resolution (Figure S15). To evaluate the spatial resolution limits of this system, a photomask featuring micrometer-scale (μm) patterned features was positioned in front of the cuvette for optical projection imaging (Figure S16). The results demonstrate that the current solution-phase system achieves only sub-mm spatial resolution due to the molecular diffusion and radiative energy transfer. This prototype of projected display presents a method of utilizing low-power NIR light to achieve a three-dimensional exhibition of perovskite QDs characterized by high-optical-property attributes.

## 3. Materials and Methods

All reagents were purchased from Innochem (Beijing, China), Sigma-Aldrich (St. Louis, MO, USA) or J&K Chemical (Beijing, China), and were used without further purification. PdTAP was obtained according to the literature procedure [46]. CsPbI_3_ QDs were prepared by the hot injection method according to the literature procedure [47].

### 3.1. Synthesis of Pd-tetrakis-5,10,15,20-(p-methoxycarbonylphenyl)-tetraanthraporphyrin (PdTAP)

To obtain a suspension, 4,11-dihydro-2H-naphtho [2,3-f]isoindole (g): f (1.46 g, 5.00 mmol, 1.00 eq.), NaOH (1.00 g, 25 mmol, 5.00 eq.), and ethylene glycol (40 mL) were added to the reaction flask. The mixture was then refluxed for 1 h under an argon atmosphere (Note: the reaction time should be limited, as prolonged heating will lead to the formation of a significant amount of by-products). After stopping the reaction and cooling to room temperature, the solvent was removed through distillation under reduced pressure. This was followed by extraction and partitioning using dichloromethane and brine. The organic phase underwent three extractions with pure dichloromethane against water, and the lower part of the funnel was subsequently collected and dried with anhydrous sodium sulphate. After removal of the solvent by spinning, the separation was carried out on the silica gel column, the eluent was dichloromethane, and the brown band on the silica gel column was collected to obtain 3.90 g of light pink solid product in 78% yield. (Sensitive to light, avoid exposure!) 1H NMR (400 MHz, CDCl_3_) δ 8.00 (s, 1H), 7.88–7.67 (m, 4H), 7.40 (dd, J = 11.6, 8.6 Hz, 2H), 6.70 (s, 2H), 4.07 (s, 4H).

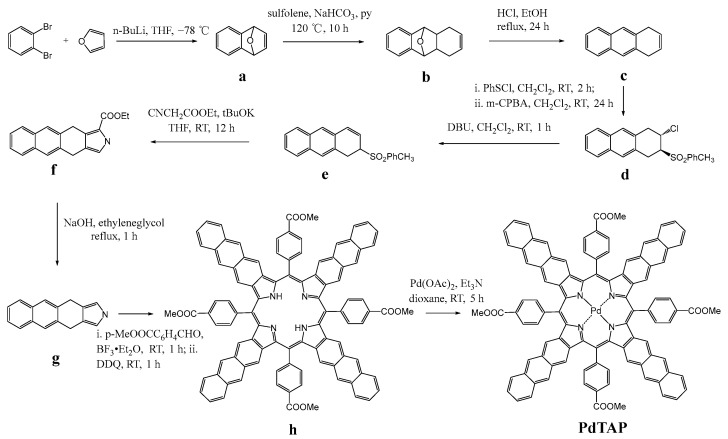

H_2_TAP (h): In a flask filled with Ar and shielded from daylight with aluminum foil, compound g (0.22 g, 1.00 mmol, 1.00 eq.) was dissolved in dry CH_2_Cl_2_ (150 mL), and p-carbobenzaldehyde (0.18 g, 1.1 mmol, 1.10 eq.) and BF_3_-Et_2_O (0.03 mL) were added sequentially. The reaction mixture was stirred at room temperature for 1 h and then a toluene solution of DDQ (0.34 g, 1.5 mmol, 1.50 eq.) was added. The mixture underwent stirring for an hour before being rinsed with 10% aqueous Na_2_SO_3_ (2 × 100 mL), 10% aqueous Na_2_CO_3_ (1 × 100 mL), and brine. Subsequently, the organic phase underwent drying with Na_2_SO_4_. Dichloromethane was used as eluent during silica gel column separation. A final product of 95.1 mg was obtained with a yield of 26.3%. (Sensitive to light, avoid exposure!) UV-Vis, Toluene, λmax nm: Soret Band 474, 511, Q-Bands 759, 830. MS MALDI-TOF: *m*/*z*: found 1447.10, calcd. for [M + H]^+^ C_100_H_63_N_4_O_8_^+^ 1447.46.

PdTAP: Two equivalents of Pd(OAc)_2_ and one drop of Et_3_N solution were added to H_2_TAP (90 mg) in dioxane solution at room temperature, and then the reaction was carried out at room temperature for 5 h. The reaction process was detected by UV-Vis spectroscopy. After the reaction was complete, the solvent was first removed by distillation under reduced pressure, and the fraction was extracted with dichloromethane and brine. Dichloromethane solutions were extracted against water three times, and the organic phase was collected from the lower layer of the partition funnel and dried and filtered with anhydrous Na_2_SO_4_. The eluent was dichloromethane for silica gel column separation, and the light pink solid product was obtained by collecting the purple band on the silica gel column. (Sensitive to light, avoid exposure!) UV-Vis, Toluene, λ_max_ nm: Soret Band 468, Q-Bands 705, 788. MS MALDI-TOF: *m*/*z*: found 1550.34, calcd. for [M + H]^+^ C_100_H_61_N_4_O_8_Pd^+^ 1551.35.

### 3.2. Synthesis of CsPbI_3_ QDs

One gram of PbI_2_ (Innochem, Beijing, China) and 50 mL of ODE (Innochem, Beijing, China) were added to a 500 mL Schlenk flask (Synthware, Chongqing, China) and stirred. The mixture was degassed under vacuum at 120 °C for 1 h. Once the degassing was completed, the flask was filled with nitrogen and held under a constant flow of nitrogen. Next, 5 mL of OA and 5 mL of OAm (Sigma-Aldrich, St. Louis, MO, USA) were injected, and the reaction system was degassed again under vacuum until PbI_2_ was fully dissolved and the solution no longer bubbled (approximately 30 min). The reactor was heated to 170 °C based on the correlation between temperature and QDs size in literature. Subsequently, the cesium oleate (Sigma-Aldrich, St. Louis, MO, USA) (~0.0625 M, 8 mL) precursor was heated to 70 °C under an N_2_ atmosphere and swiftly introduced into the reactor. The solution was rapidly quenched by immersing the Schlenk bottle in an ice water bath approximately five seconds after the introduction was completed.

The solution was split into eight equal parts and transferred to eight centrifuge tubes. Each tube had a volume of approximately 7.5 mL. MeOAc (J&K Chemical, Beijing, China), in a ratio of 1:3 to the reaction solution, was added (i.e., 20 mL of MeOAc). The mixture was centrifuged at 8000 rpm for 5 min. After this process, the supernatant was carefully decanted, and the precipitate was dispersed in 3 mL of hexane. Then, an equal amount of MeOAc was added to the mixture. The mixture was centrifuged again at 8000 rpm for 2 min, and the supernatant was discarded afterwards. The reaction mixture must be promptly transferred to 20 mL of hexane and centrifuged at 4000 rpm for 5 min. Subsequently, the colloidal CsPbI_3_ quantum dots solution needs to be kept in the dark at 4 °C for 48 h to allow the surplus Cs-oleate and Pb-oleic acid to precipitate. Then, the solution must be subjected to another centrifugation at 4000 rpm for 5 min, and the supernatant collected.

### 3.3. Instrumentation

^1^H NMR spectra were collected with a Bruker Avance P-400 (400 MHz) spectrometer (Bruker, Billerica, MA, USA), with tetramethylsilane used as an internal standard. Bruker Autoflex III Smartbeam MALDI-TOF MS (Bruker, Billerica, MA, USA) was employed in the mass spectrometry. JEM-2100F (JEOL Ltd., Akishima, Japan) was used to obtain TEM images, with samples positioned on holey copper grids. A Shimadzu UV-2550PC spectrophotometer (Shimadzu, Kyoto, Japan) and a Hitachi F-4600 spectrometer (Hitachi, Chiyoda, Japan) were used to record UV-Vis absorption spectra and emission spectra, respectively. The lifetimes of fluorescence in the examined solutions were measured utilizing the Edinburgh FLS1000 spectrometer (Edinburgh Instruments, Livingston, UK). The time-correlated single photon counting (TCSPC) method was used, exciting the solutions with a nanosecond pulse laser (EPL 375) (Edinburgh Instruments, Livingston, UK) at 375 nm.

Upconversion emissions were recorded using an integrating sphere from Labsphere (Sutton, UK), which was combined with a Princeton Instrument Acton SP2500 spectrograph and a SPEC-10 liquid-nitrogen-cooled CCD (Acton, MA, USA). An 808 nm laser (MDL-H-808nm-5W, Changchun New Industry Optoelectronic Technology Co., Ltd., Changchun, China) was used as the incident light for the upconversion tests.

### 3.4. Measurement

#### 3.4.1. Upconversion

All samples for upconversion experiments were prepared in oxygen-free conditions. Prior to determining the upconversion luminescence quantum yield, the sample must undergo bubbling and de-oxygenation in an argon atmosphere of high-purity (>99.999%) for 30 min.

Owing to the paucity of NIR dyes and restricted test conditions for NIR luminescence quantum yields, it is inappropriate to utilize relative upconversion quantum yields ascertained by the reference method in conventional solutions for NIR excited systems. To measure the upconversion quantum yield of NIR excited systems, this study employed the rubrene toluene solution with 532 nm excitation as a reference (98% quantum yield). The calculated equation is as follows:

(1) $$\Phi_{\mathrm{UC}}=\Phi_{ref}\times \frac{\left(\mathrm{photons}\;\mathrm{absorbed}\;\mathrm{by}\;\mathrm{reference}\right)}{\left(\mathrm{photons}\;\mathrm{absorbed}\;\mathrm{by}\;\mathrm{UC}\;\mathrm{sample}\right)}\times \frac{\mathrm{signal}(\mathrm{UC}\;\mathrm{sample})}{\mathrm{signal}(\mathrm{reference})}$$

(2) $$\mathrm{Photons}\mathrm{absorbed}/s=\frac{Laser\;Power}{\frac{hc}{\lambda }}\left(1-10^{-Abs}\right)$$

where Φ_UC_ is the upconversion quantum yield, Φ_ref_ is the quantum yield of the reference sample, *h* is the Planck constant, *c* is the speed of light, and *Abs* is the absorption of the sample.

#### 3.4.2. Calculation of Spectral Overlap Integral

The spectral overlap integral was estimated by following the well-accepted equation, which takes account of the donor fluorescence and acceptor absorption properties, respectively [48]:

(3) $$J\left(\lambda \right)=\int_{0}^{\infty }F_{D}\left(\lambda \right)\epsilon_{A}\left(\lambda \right)\lambda^{4}d\lambda \;\;\;M^{-1}{\mathrm{cm}}^{-1}{\mathrm{nm}}^{4}$$

where *F_D_*(*λ*) is the normalized fluorescence intensity of donor at a particular wavelength (*λ*) and *ϵ_A_* is the molar extinction coefficient of the acceptor at a particular wavelength.

#### 3.4.3. Volumetric Display

All samples for upconversion experiments were prepared in oxygen-free conditions. In projected display experiments, excitation optical power densities above a threshold are used to activate the upconversion regime with an excitation wavelength of 808 nm. Various excitation source shapes can be realized by filtering the light through an aperture. Volume display through the quadratic nature of upconversion can be realized with 20× objective of focusing.

## 4. Conclusions

In summary, CsPbI_3_ QDs have been utilized as the energy collector to harvest triplet fusion upconversion emission from the rubrene, entitling CsPbI_3_ QDs excitation with NIR photons. Both steady-state and time-resolved photoluminescence experiments indicate the predominance of radiative energy transfer in the organic–inorganic pair of rubrene and the QDs. Significantly, this process does not compromise the inherent photophysical processes within the organic–inorganic hybrid system. This hybrid system requires an excited power density of about 134 mW/cm^2^ to activate. The upconversion luminescence quantum yield of this system is estimated to be 0.48% with NIR excitation at a power density of 13.1 W/cm^2^. This result not only facilitates the first use of perovskite QDs in projected volumetric displays through NIR light excitation, but also underscores the potential for synergizing organic upconversion to energize perovskite QDs. This study offers a potential design for the coupling of perovskite QDs and organic materials, holding implications for solar energy conversion and lighting.

## Data Availability

Data are contained within the article and Supplementary Materials.

## Supplementary Materials

The following supporting information can be downloaded at: https://www.mdpi.com/article/10.3390/molecules30112273/s1. Figure S1: Schematic illustration of the experimental setup for the characterization of TTA-UC emission; Figure S2: UV-Vis absorption spectra of H_2_TAP and PdTAP; Figures S3 and S4: Mass spectrum of H_2_TAP and PdTAP; Figure S5: Phosphorescence spectrum of PdTAP; Figure S6: Transmission electron micrograph of CsPbI_3_ QDs; Figure S7: PLQY of various CsPbI_3_ QDs concentrations; Figure S8: Phosphorescence lifetime of Rub/CsPbI_3_ QDs solution at 1150 nm under different conditions; Figure S9: Upconversion luminescence spectra of CsPbI_3_ QDs under various contrast conditions; Figure S10: (a) Absorption spectra of rubrene (500 μM) doped with different concentrations of CsPbI_3_ QDs in toluene. (b) Normalized excitation spectra of rubrene (500 μM) doped with different concentrations of CsPbI_3_ QDs in toluene at 675 nm; Figure S11: Upconversion luminescence spectra of (a) PdTAP/rubrene and (b) PdTAP/rubrene/CsPbI_3_ QDs at different excitation light power densities. (c) Double logarithmic curve of the corresponding upconversion system; Figure S12: Decay kinetics of the CsPbI_3_ QDs (1 mg/mL) at 670 nm; Figure S13: (a) Delayed fluorescence lifetime at different wavelengths without CsPbI_3_ QDs and doped with (b) CsPbI_3_ QDs; Figure S14: (a) Absorption and (b) upconversion emission spectra of the PdTAP/rubrene/CsPbI3 QDs hybrid system in toluene under 808 nm CW laser excitation (13.1 W/cm^2^) at different irradiation durations; Figure S15: Quadratic process results from triplet fusion upconversion by exciting the sensitizer at 808 nm, and the red light is generated only at the focal spot upon (a) low excitation power intensity and (b) high excitation power intensity; Figure S16: (a) Optical-projection photomask. (b) and (c) Luminescent pattern images of the PdTAP/rubrene/CsPbI_3_ QDs solution illuminated by NIR light transmitted through the photomask; Table S1: Summary of the recently reported work on upconversion-activated perovskites. References [16,47,49,50,51,52,53,54,55,56,57] is cited in the Supplementary Materials.
