# Supplementary material for: Activation of Perovskite Nanocrystals for Volumetric Displays Using Near-Infrared Photon Upconversion by Triplet Fusion
Source: Molecules. 2025 May 22;30(11):2273. doi: 10.3390/molecules30112273 (PMC12156221; doi:10.3390/molecules30112273)
Supplement: Supplementary file 1 [file molecules-30-02273-s001.zip › molecules-3584201-supplementary.pdf]

Supplementary Materials for

Activation of Perovskite Nanocrystals for Volumetric Displays Using Near-Infrared Photon Upconversion by Triplet Fusion

Yu Hu [1,2], Guiwen Luo [1,*], Pengfei Niu [1], Ling Zhang [1], Tianjun Yu [1], Jinping Chen [1], Yi Li [1,2] and Yi Zeng [1,2,*]

1 Key Laboratory of Photochemical Conversion and Optoelectronic Materials, Technical Institute of Physics and Chemistry, Chinese Academy of Sciences, Beijing 100190, China; huyu20@mails.ucas.ac.cn (Y.H.); niupengfei18@mails.ucas.ac.cn (P.N.); zhangling21@mails.ucas.ac.cn (L.Z.); tianjun_yu@mail.ipc.ac.cn (T.Y.); chenjp@mail.ipc.ac.cn (J.C.); yili@mail.ipc.ac.cn (Y.L.)

2 University of Chinese Academy of Sciences, Beijing 100049, China

* Correspondence: luoguiwen17@mails.ucas.ac.cn (G.L.); zengyi@mail.ipc.ac.cn (Y.Z.)

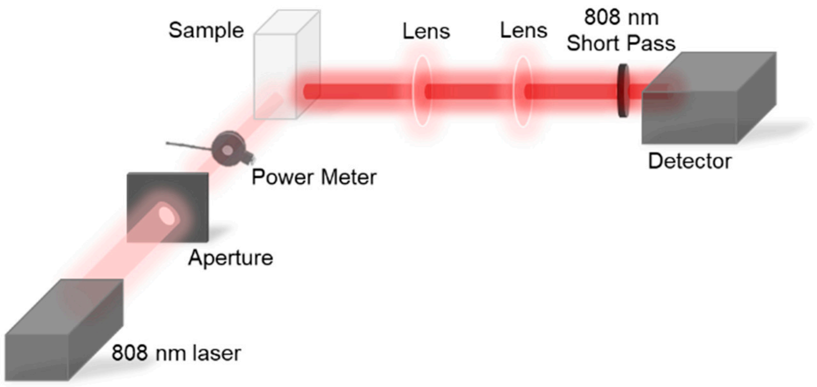

Figure S1. Schematic illustration of the experimental setup for the characterization of TTA-UC emission.

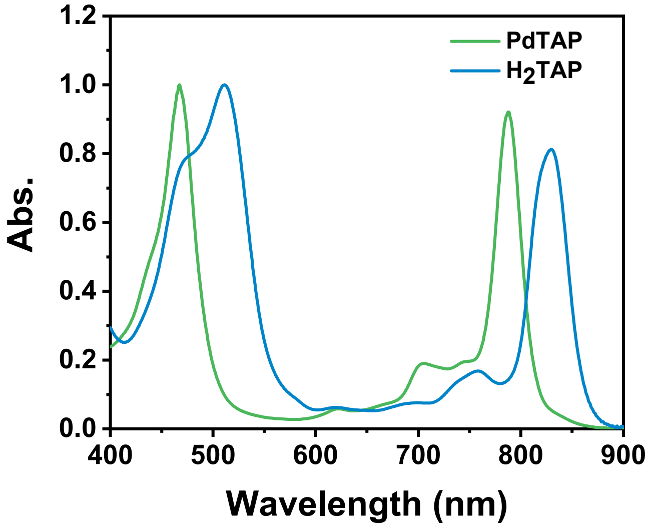

Figure S2. UV-Vis absorption spectra of H_2TAP and PdTAP in toluene.

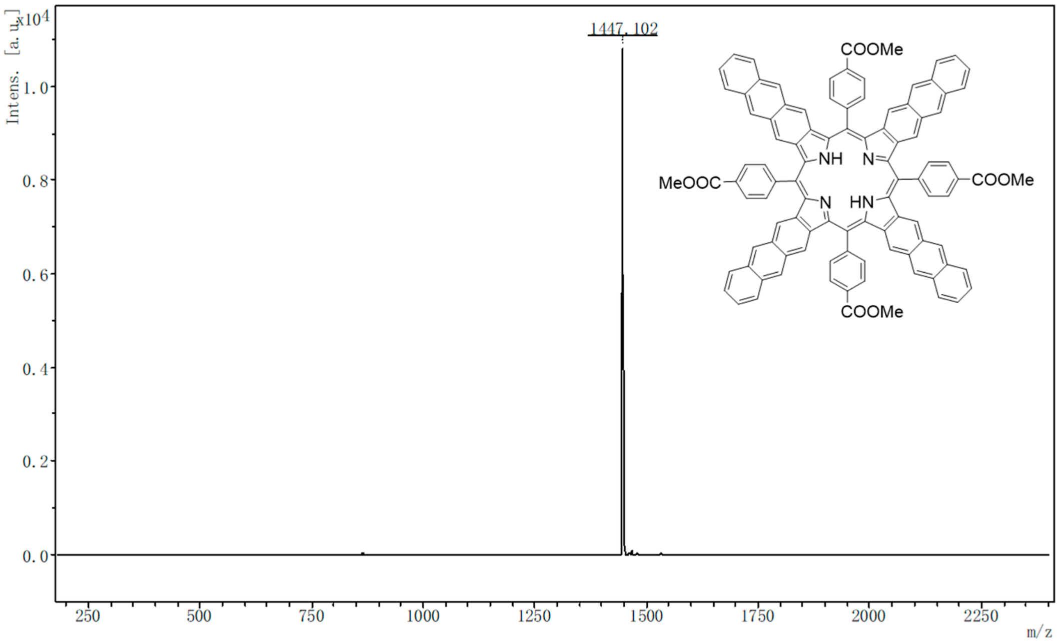

Figure S3. Mass spectrum of H_2TAP.

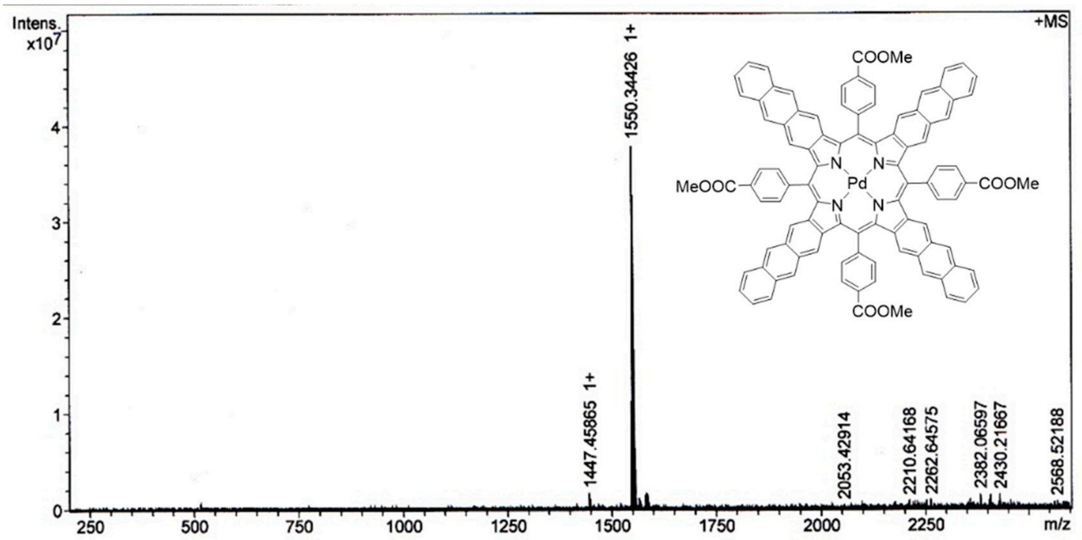

Figure S4. Mass spectrum of PdTAP.

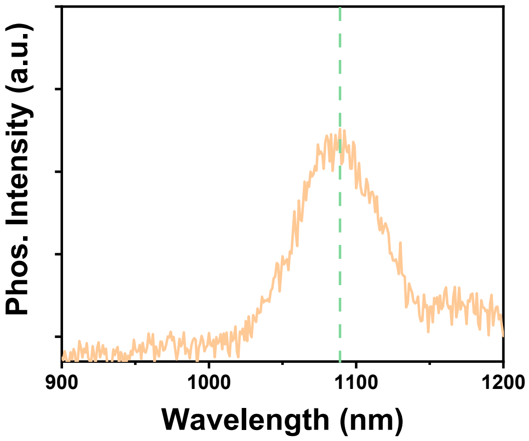

Figure S5. Phosphorescence spectrum of PdTAP (λ_{ex} = 808 nm).

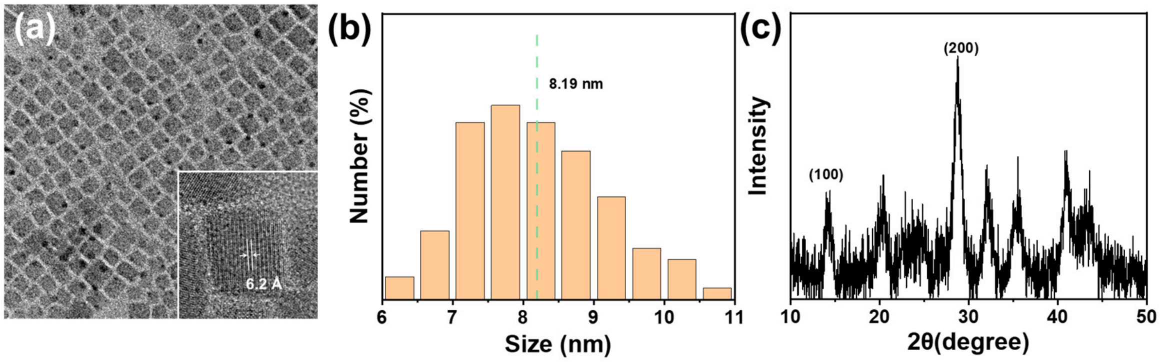

Figure S6. (a) Transmission electron micrograph (TEM) of $CsPbI_3$ quantum dots. (b) Particle size distribution of $CsPbI_3$ quantum dots. (c) XRD patterns of $CsPbI_3$ QDs, confirming that they crystallize in the cubic phase of $CsPbI_3$[1].

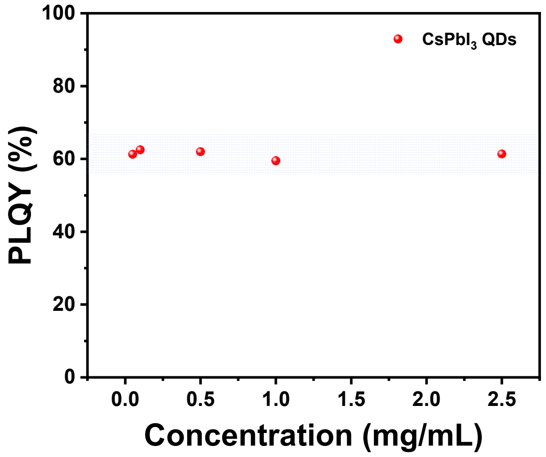

Figure S7. PLQY of various $CsPbI_3$ QDs concentrations (λ_{ex} = 500 nm).

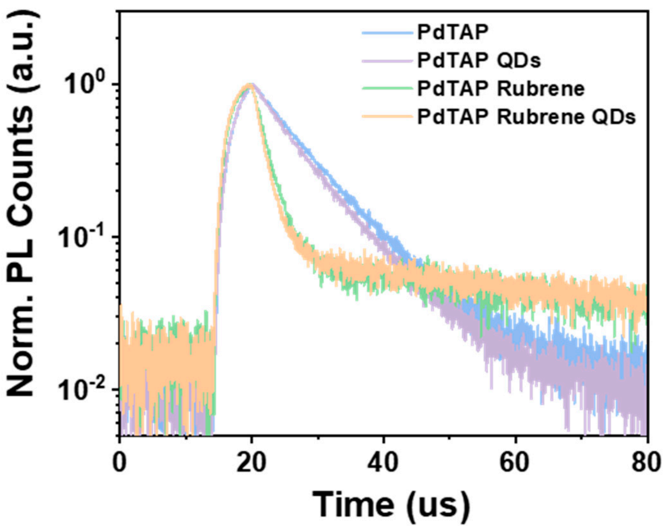

Figure S8. Phosphorescence lifetime of hybrid system at 1150 nm under different conditions (λ_{ex} = 808 nm).

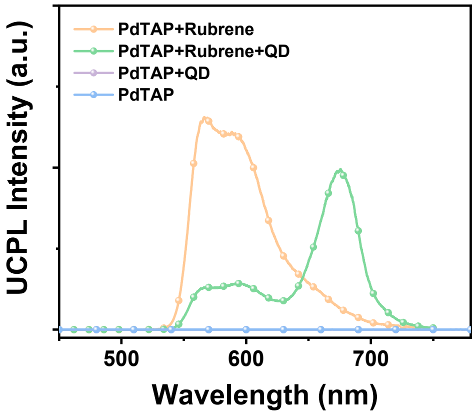

Figure S9. Upconversion luminescence spectra of the hybrid system under various contrast conditions (λ_{ex} = 808 nm).

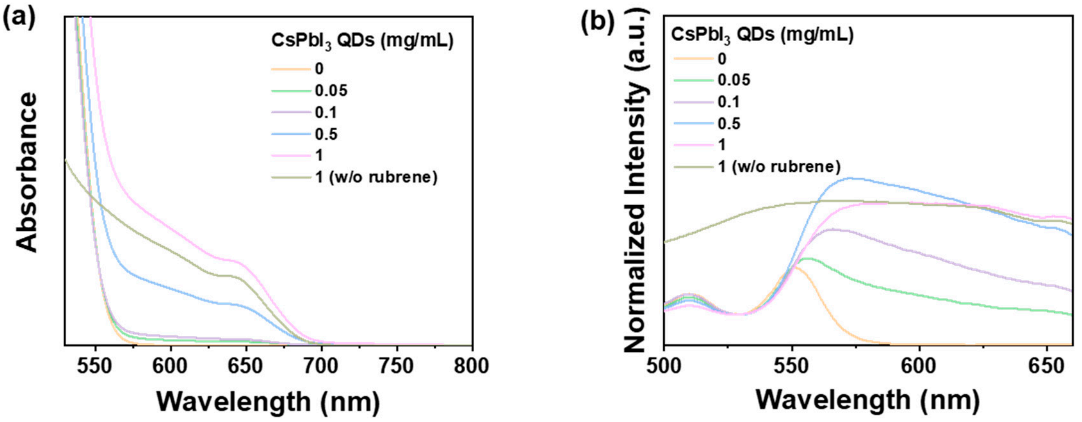

Figure S10. (a) Absorption spectra of rubrene (500 μM) doped with different concentrations of $CsPbI_3$ QDs in toluene. (b) Normalized excitation spectra of rubrene (500 μM) doped with different concentrations of $CsPbI_3$ QDs in toluene solution at 675 nm.

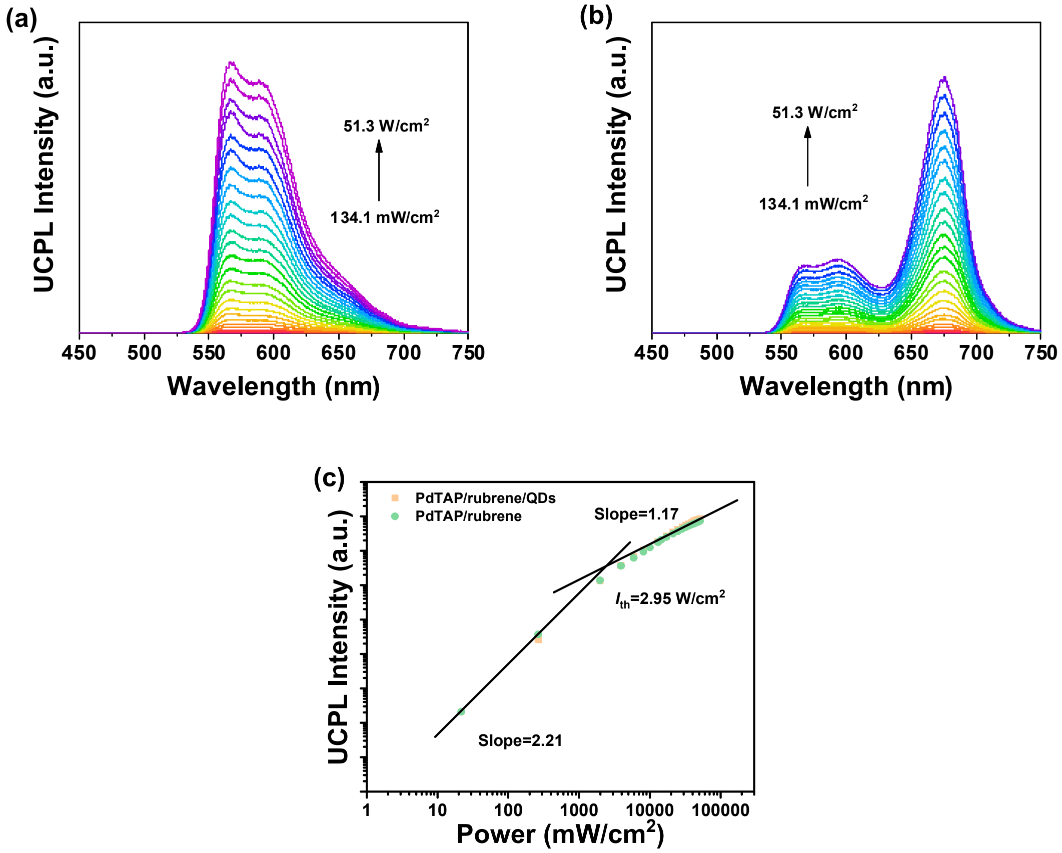

Figure S11. Upconversion luminescence spectra of (a) PdTAP/rubrene and (b) PdTAP/rubrene/$CsPbI_3$ QDs at different excitation power densities. (c) Double logarithmic curve of the corresponding upconversion system (λ_{ex} = 808 nm).

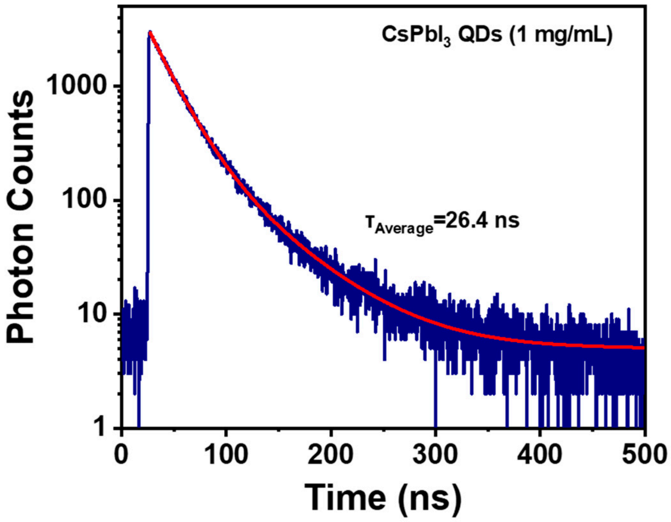

Figure S12. Decay kinetics of the $CsPbI_3$ QDs (1 mg/mL) at 670 nm (λ_{ex} = 510 nm).

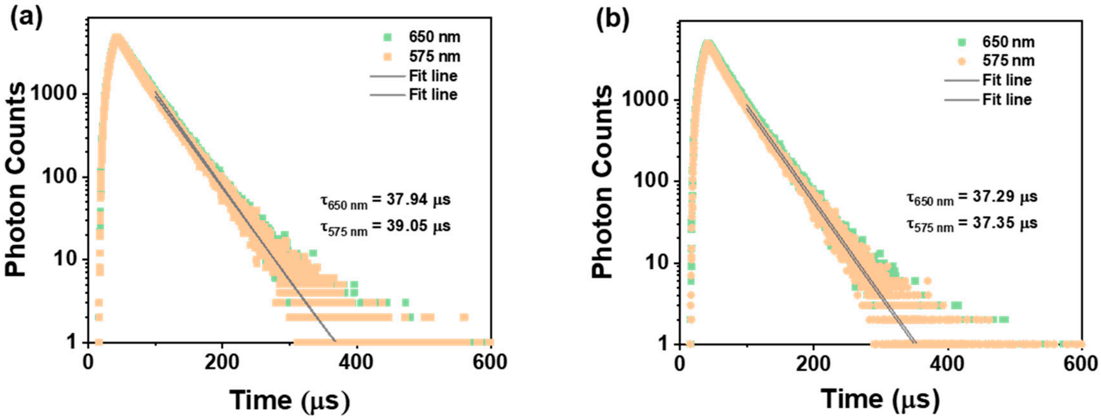

Figure S13. (a) Delayed upconversion emission lifetime at different wavelengths without $CsPbI_3$ QDs and doped with (b) $CsPbI_3$ QDs (λ_{ex} = 808 nm).

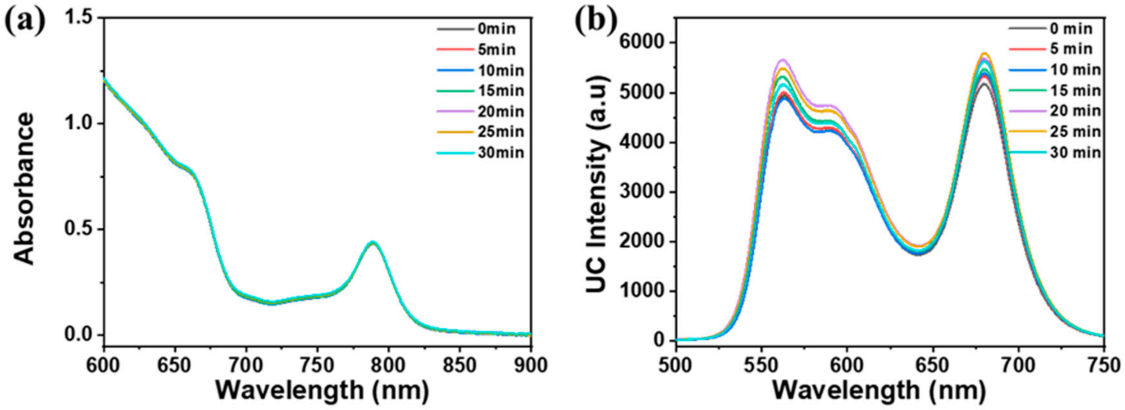

Figure S14. (a) Absorption and (b) upconversion emission spectra of the PdTAP/rubrene/$CsPbI_3$ QDs hybrid system in toluene under 808 nm CW laser excitation (13.1

W/cm^2) at different irradiation durations.

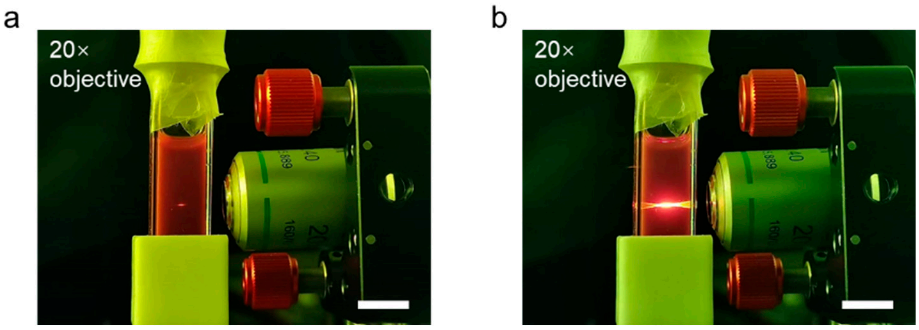

Figure S15. The quadratic process results from triplet fusion upconversion by exciting the sensitizer at 808 nm, and the red light is generated only at the focal spot upon (a) low excitation power intensity and (b) high excitation power intensity. The scale bars denote 1 cm.

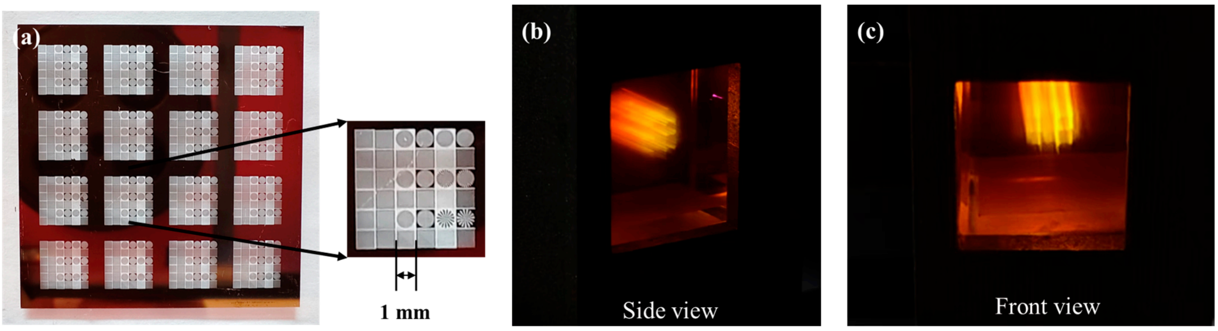

Figure S16. (a) Optical-projection photomask. (b) and (c) Luminescent pattern images of the PdTAP/rubrene/$CsPbI_3$ QDs solution illuminated by NIR light transmitted through the photomask.

Table S1. Summary of recently reported work on upconversion-activated perovskites.

**Materials**	**Activated power density**	**Upconversion quantum yield**	**Reference**
UCNPs-Organolead halide perovskites	13.9 W/cm^2	—	*Adv. Funct. Mater.* **2018,** *28,* 1801782. [2]
UCNPs-PeQDs	36.8 W/cm^2	—	*Adv. Mater.* **2020,** *32,* 2000820. [3]
UCNPs-PeQDs	—	0.32 %	*New J. Chem.* **2018,** *42,* 12353. [4]
UCNPs-PeQDs	~3.0 W/cm^2	0.33-0.45% (±0.07-0.13%)	*Nat. Commun.* **2018,** *9,* 3462. [5]
β-$NaYF_4$:30%Yb, 0.2%Tm NPs-QDs	2.45 W/cm^2	—	*J. Mater. Chem. C.* **2019,** *7,* 3751-3755. [6]
$Yb^{3+}/Er^{3+}/Bi^{3+}$: $Cs_2Ag_{0.6}Na_{0.4}InCl_6$	4 W/cm^2	—	*Adv. Mater.* **2020,** *32,* 2004506. [7]

AuNRs-UCNPs/PeQDs-Ag film	1.45 W/cm^2	—	*J. Mater. Chem. C.* **2022,** *10*, 532-541. [8]
Tm:KYb$_2$F$_7$@glass-CsPbX$_3$ PeNCs@glass	3 W/cm^2	—	*Chem. Eng. J.* **2020,** *395,* 125214. [9]
Heterostructured CsPbBr$_3$-NaYF$_4$:Yb,Tm	—	0.223%	*Nat. Commun.* **2021,** *12,* 219. [10]
UCNPs-PeQDs	50 W/cm^2	—	*Adv. Mater.* **2021,** *33,* 2101852. [11]
**Triplet fusion UC-PeQDs**	**134 mW/cm^2**	**0.48%**	**This work**

References

1. Swarnkar, A.; Marshall, A.R.; Sanehira, E.M.; Chernomordik, B.D.; Moore, D.T.; Christians, J.A.; Chakrabarti, T.; Luther, J.M. Quantum dot-induced phase stabilization of α-CsPbI3 perovskite for high-efficiency photovoltaics. *Science* **2016**, *354*, 92–95.
2. Yang, B.; Wang, Y.; Wei, T.; Pan, Y.; Zhou, E.; Yuan, Z.; Han, Y.; Li, M.; Ling, X.; Yin, L.; et al. Solution-Processable Near-Infrared–Responsive Composite of Perovskite Nanowires and Photon-Upconversion Nanoparticles. *Adv. Funct. Mater.* **2018**, *28*, 1801782.
3. Yang, X.; Zhou, M.; Wang, Y.; Duan, P. Electric-field-regulated energy transfer in chiral liquid crystals for enhancing upconverted circularly polarized luminescence through steering the photonic bandgap. *Adv. Mater.* **2020**, *32*, 2000820.
4. Rao, M.; Fu, J.; Wen, X.; Sun, B.; Wu, J.; Liu, X.; Dong, X. Near-infrared-excitable perovskite quantum dots via coupling with upconversion nanoparticles for dual-model anti-counterfeiting. *New J. Chem.* **2018**, *42*, 12353.
5. Zheng, W.; Huang, P.; Gong, Z.; Tu, D.; Xu, J.; Zou, Q.; Li, R.; You, W.; Bünzli, J.-C.G.; Chen, X. Near-infrared-triggered photon upconversion tuning in all-inorganic cesium lead halide perovskite quantum dots. *Nat. Commun.* **2018**, *9*, 3462.
6. Ma, J.; Wu, H.; Qiu, J.; Wang, J.; Wang, Q.; Yang, Y.; Zhou, D.; Han, J. NIR-excited all-inorganic perovskite quantum dots (CsPbBr 3) for a white light-emitting device. *J. Mater. Chem. C* **2019**, *7*, 3751–3755.
7. Zeng, Z.; Huang, B.; Wang, X.; Lu, L.; Lu, Q.; Sun, M.; Wu, T.; Ma, T.; Xu, J.; Xu, Y.; et al. Multimodal luminescent Yb3+/Er3+/Bi3+-doped perovskite single crystals for X-ray detection and anti-counterfeiting. *Adv. Mater.* **2020**, *32*, 2004506.
8. Kim, M.; Kim, Y.; Kim, K.; Huang, W.T.; Liu, R.S.; Hyun, J.K.; Kim, D.H. Gap surface plasmon-enhanced photoluminescence from upconversion nanoparticle-sensitized perovskite quantum dots in a metal–insulator–metal configuration under NIR excitation. *J. Mater. Chem. C* **2022**, *10*, 532–541.
9. Lin, J.; Yang, C.; Huang, P.; Wang, S.; Liu, M.; Jiang, N.; Chen, D. Photoluminescence tuning from glass-stabilized CsPbX3 (X= Cl, Br, I) perovskite nanocrystals triggered by upconverting Tm: KYb2F7 nanoparticles for high-level anti-counterfeiting. *Chem. Eng. J.* **2020**, *395*, 125214.
10. Ruan, L.; Zhang, Y. NIR-excitable heterostructured upconversion perovskite nanodots with improved stability. *Nat. Commun.* **2021**, *12*, 219.
11. Xie, L.; Hong, Z.; Zan, J.; Wu, Q.; Yang, Z.; Chen, X.; Ou, X.; Song, X.; He, Y.; Li, J.; et al. Broadband detection of X-ray, ultraviolet, and near-infrared photons using solution-processed perovskite–lanthanide nanotransducers. *Adv. Mater.* **2021**, *33*, 2101852.
